# Predictive value of lymphocyte-to-C-reactive protein ratio for left ventricular thrombus in patients with ST-segment elevation myocardial infarction

**DOI:** 10.3389/fcvm.2025.1465350

**Published:** 2025-04-24

**Authors:** Xinjia Du, Jiahua Liu, Zeqing Zhang, Yanfei Ren, Lei Chen, Yuan Lu, Zhuoqi Zhang

**Affiliations:** ^1^ Department of Cardiology, The Affiliated Hospital of Xuzhou Medical University, Xuzhou, China; ^2^ Department of Cardiology, Shanghai Tenth People’s Hospital, Tongji University School of Medicine, Shanghai, China

**Keywords:** inflammation, left ventricular thrombus, myocardial infarction, cardiovascular magnetic resonance, lymphocyte to C-reactive protein ratio

## Abstract

**Background and purpose:**

Current evidence suggested a correlation between inflammation and Left Ventricular Thrombus (LVT). The lymphocyte to C-reactive protein ratio (LCR) has been established as be a reliable inflammation marker and is associated with the prognosis of patients with ST-segment elevation myocardial infarction (STEMI). However, its relationship with the occurrence of LVT remains unclear. This study aims to evaluate the effectiveness of LCR in predicting LVT in patients with STEMI after undergoing primary percutaneous coronary intervention (pPCI).

**Methods:**

A total of 564 STEMI patients who underwent pPCI at the Affiliated Hospital of Xuzhou Medical University from September 2019 to June 2024 were included. Cardiac magnetic resonance imaging (CMR) was used to assess myocardial infarction characteristics and the presence of LVT. The definition of LCR is the lymphocyte to C-reactive protein ratio.

**Results:**

Out of 564 patients, 57 were diagnosed with LVT. The median time for CMR testing was 5 (4, 6) days. Univariate regression analysis showed significant differences in left ventricular ejection fraction (LVEF), peak N-terminal pro B-type natriuretic peptide (peak NT-proBNP), peak high-sensitivity troponin T (peak hsTnT), LCR, Late Gadolinium Enhancement% (LGE%), and Microvascular Obstruction% (MVO%) (*p* < 0.05). Multivariate regression analysis indicated that LCR was an independent predictor for LVT (*P* = 0.007, OR: 0.001 95% CI: 0.00–0.123). Receiver operating characteristic (ROC) curve analysis showed that LCR has good predictive ability for LVT (Area under the curve: 0.704, *p* < 0.001). Integration of integral LCR could significantly improve the discrimination and reclassification accuracy for LVT after STEMI (NRI = 0.517, IDI = 0.030; *p* < 0.001).

**Conclusion:**

Lower LCR is independently associated with the risk of LVT in patients with STEMI after pPCI. Integration of LCR can significantly improve the risk model for LVT.

## Introduction

Cardiovascular diseases pose a major threat to humans (1, 2). Acute coronary artery disease manifests as an ST-segment elevation myocardial infarction (STEMI). Patients with STEMI continue to experience significant rates of impairment and mortality, even with ongoing optimization of treatment strategies (2). Irreversible myocardial necrosis and its related consequences are among the possible causes. Following STEMI, up to 12% of patients may develop left ventricular thrombus (LVT) (3). Mechanistically, LVT is caused by endothelial damage because of inflammatory responses, blood stasis from abnormal wall motion following STEMI, and a hypercoagulable state (4). LVT is associated with major adverse cardiac events and poor prognosis in patients (5–7). Therefore, identifying additional risk factors for LVT will help identify high-risk patients early, allowing for timely intervention and improved prognosis in patients with STEMI.

Commonly used diagnostic methods for LVT include transthoracic echocardiography (TTE) and cardiac magnetic resonance (CMR) imaging, among which CMR is the most accurate imaging modality for detecting LVT (8). CMR outperforms TTE in detecting smaller, layered LVTs (9). A recent meta-analysis of 2,072 patients with STEMI reported that among 431 patients with both CMR and TTE data, the sensitivity of TTE was only 29%, possibly because of poor visualization of the apical region, limiting its diagnostic utility for LVT (9–11). PInflammatory markers, such as high-sensitivity C-reactive protein (CRP), interleukin-6, fibrinogen, and the systemic inflammation index can predict LVT (5, 12). Recently, the lymphocyte-to-CRP ratio (LCR), a new and valuable inflammatory biomarker, has emerged as a useful tool for predicting outcomes and guiding treatment decisions in various diseases, including cancer (7, 13–16), the severity of myocardial injury, and the progression of COVID-19 (17). A previous study showed that LCR is an independent predictor of short- and long-term major adverse cardiac events after acute myocardial infarction (18). In addition, LCR has been associated with new-onset atrial fibrillation in patients with STEMI undergoing primary percutaneous coronary intervention (pPCI) (19). However, the relation between LCR and LVT in patients with STEMI remains unclear. This study aimed to investigate the predictive value of LCR for LVT after pPCI in patients with STEMI.

## Materials and methods

### Study population

**Figure 1:**
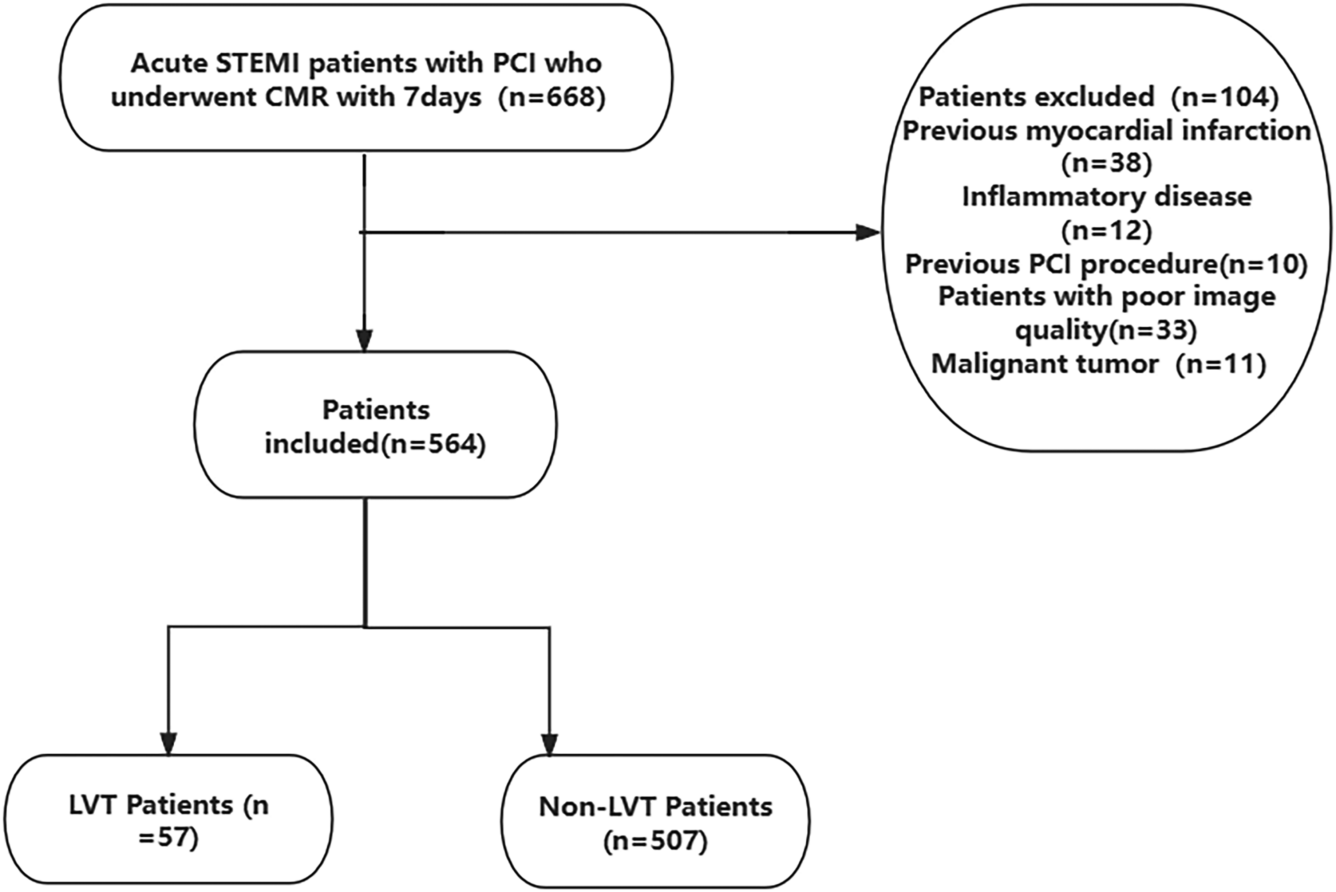
Study flowchart.

This was a single-center retrospective study that consecutively included STEMI (2) patients who underwent pPCI at Xuzhou Medical University Affiliated Hospital from September 2019 to June 2024. Inclusion criteria: successful pPCI within 12 hours of symptom onset (TIMI ≥ 2), estimated Glomerular filtration rate (eGFR) > 30 ml/min/1.73m^2^. Exclusion criteria were a history of malignancy, inflammatory disease, previous PCI, previous myocardial infarction and poor quality of CMR. The requirement for signed written consent was waived owing to the low risk to the patient by the relevant IRB regulatory guidelines. This study was approved by the Ethics Committee of Xuzhou Medical University Affiliated Hospital. The ethics approval number is XYFY2024-KL207-01. The inclusion and exclusion criteria are shown in Figure 1.

### Clinical data collection

**Figure 2:**
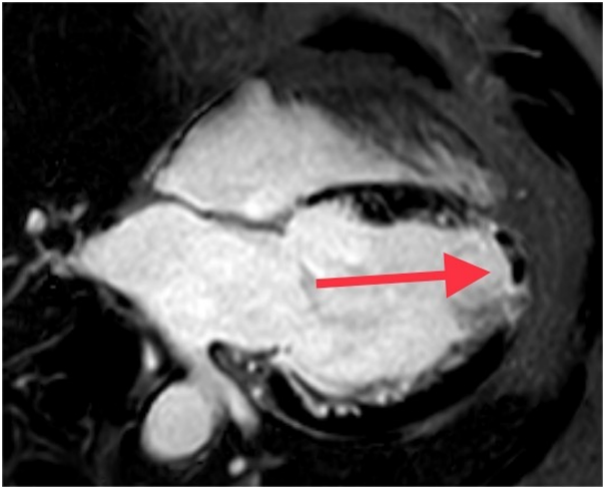
Late gadolinium enhancement cardiovascular magnetic resonance image demonstrating an apical left ventricular (LV) thrombus (red arrow, black filling defect). LGE, Late gadolinium enhancement; LVT, left ventricular thrombus.

Patient data were collected from the hospital's medical record system, including name, height, weight, age, smoking history, hypertension, diabetes, medication use, systolic and diastolic blood pressure, heart rate, and Killip class at admission. Routine blood tests were conducted during hospitalization, and values for various markers were recorded, including lymphocytes, CRP, hs-TnT, and NT-proBNP were measured multiple times during hospitalization. The peak values during hospitalization were used for this study. Based on the results of the coronary angiography, we documented the Infarct-related arteries (IRA). All patients who underwent pPCI had CMR using a 3.0 T scanner (Philips, Netherlands) within 5(4, 6) days (Median time of CMR) during hospitalization. Subsequently, image analysis was performed using CVI42 (version 5.13.5 Circle Cardiovascular Imaging Canada). To ensure accuracy, the tracking performance was checked after automatic analysis and manually adjusted if necessary. On the short-axis LGE images, the endocardial and epicardial borders were manually traced to delineate the myocardium. LGE is defined as the mass of myocardial infarction, expressed as the percentage of the hyperenhanced myocardial area relative to the total myocardial mass, usually denoted as LGE%. Similarly, microvascular obstruction (MVO) was assessed on LGE images as a low-signal region surrounded by high-signal myocardial tissue, and the mass was quantified by manually delineating the low-signal region within the enhanced area (20), it is usually expressed as MVO%, which refers to the percentage of the low-signal region within the enhanced area relative to the total myocardial mass. The CMR diagnosis of LVT was determined by the presence of a mass that doesn't improve within the left ventricular cavity, which is distinct from the endocardium, papillary muscles, chordae, trabeculae, or artifact (11, 21) (Figure 2).

### Statistical analysis

Statistical analysis was conducted using SPSS version 26.0 (Inc, Chicago, IL, USA) and R 4.1.2 (https://cran.r-project.org). The Kolmogorov–Smirnov test was used to evaluate the normality of data distribution. Normally distributed continuous variables were expressed as mean ± standard deviation and analyzed using Student's *t*-test. Non-normally distributed continuous variables were expressed as median (Q1, Q3) and analyzed using the Mann–Whitney *U*-test. Categorical variables were expressed as frequencies and percentages and analyzed using the chi-square test. Correlation of LCR with other variables related to LVT using Spearman analysis. All variables were included in the univariate logistic regression analysis, and variables with *P* < 0.05 were included in the multivariate logistic regression analysis. A forward stepwise multivariate logistic regression analysis was used to determine the correlation between LCR and LVT. Receiver Operating Characteristic (ROC) curve was used to evaluate the sensitivity and specificity of LCR in predicting LVT, and the optimal cut-off value of LCR for predicting LVT was obtained. The net reclassification index (NRI) and integrated discrimination improvement (IDI) were used to evaluate the improvement in the discrimination and reclassification abilities of the model with LCR. A two-tailed *P* < 0.05 was considered statistically significant.

## Results

### Baseline data comparison between groups

This study included a total of 564 patients who underwent cardiac magnetic resonance (CMR) imaging during hospitalization. The mean age of the patients was 57.66 ± 11.87 years. A total of 77.0% of patients were men, 52.0% had hypertension, 52.0% had dyslipidemia, 30.0% were current or previous smokers, 29.0% had diabetes mellitus, and 10.0% had a family history of CAD, 9.93% of the patients had a history of stroke. 57 patients (10.1%) were characterized with LVT, while 507 patients (89.9%) did not have LVT. As shown in Table 1, among the laboratory indicators, compared to the non LVT group, the LVT group had significantly higher levels of peak hs-TnT (*p* = 0.030), and peak NT-proBNP (*p* = 0.006), LGE% (*p* = 0.003), MVO% (*p* = 0.007) and lower levels of LVEF (*p* = 0.009), and LCR (*p* < 0.001). For cardiac angiography-related indicators, the differences were statistically significant for IRA-LAD (*p* = 0.008) and IRA-RCA (*p* = 0.001).

**Table 1:** Baseline data comparison between groups.

Variables	Non LVT (*n* = 507)	LVT (*n* = 57)	*P*
Male, n(%)	434 (85.77)	53 (92.98)	0.131
Age, (years)	57.68 ± 11.98	57.54 ± 10.96	0.936
BMI, (kg/m^2^)	25.86 ± 3.68	26.12 ± 2.78	0.608
Systolic blood pressure, (mm/Hg)	126.65 ± 18.78	127.54 ± 20.18	0.736
Diastolic blood pressure, (mm/Hg)	80.09 ± 12.75	82.56 ± 12.98	0.167
Heart rate, (times/min)	79.11 ± 12.03	80.14 ± 16.83	0.656
Peak hsTnT, (ng/L)	3,259.00 (1,508.0, 6,374.0)	4,841 (2,011.0, 10,000.0)	0.030
Peak NTproBNP, (pg/ml)	1,274.65 (720.1, 2,195.5)	1,942.00 (963.0, 2,843.0)	0.006
TC, (mmol/L)	1.68 ± 1.19	1.98 ± 1.69	0.085
TG, (mmol/L)	4.38 ± 1.07	4.33 ± 0.99	0.739
LDL-C, (mmol/L)	2.81 ± 1.01	2.70 ± 0.78	0.407
HDL-C, (mmol/L)	0.97 ± 0.25	1.02 ± 0.54	0.445
Fasting blood glucose, (mmol/L)	6.61 ± 2.53	7.14 ± 2.91	0.138
eGFR, (ml/min/1.73 m^2^)	107.38 ± 15.66	108.42 ± 16.53	0.637
LVEF, (%)	52.53 ± 6.37	50.21 ± 6.14	0.009
PLT, (*10^9^/L)	217.04 ± 60.25	219.12 ± 62.29	0.806
LCR	0.05 (0.02, 0.13)	0.02 (0.01, 0.05)	<.001
LGE%	30.03 ± 17.97	37.47 ± 18.15	0.003
MVO%	0.00 (0.00, 1.77)	1.43 (0.00, 4.99)	0.007
ESVI, (ml/m^2^)	33.22 ± 12.61	31.94 ± 10.37	0.389
EDVI, (ml/m^2^)	24.28 ± 15.56	23.25 ± 14.00	0.631
Smoking, *n* (%)	261 (51.48)	33 (57.89)	0.358
Hypertension, *n* (%)	228 (44.97)	28 (49.12)	0.550
Diabetes, *n* (%)	123 (24.26)	20 (35.09)	0.075
Stroke, *n* (%)	53 (10.45)	3 (5.26)	0.214
Killip class ≥2, *n* (%)	30 (5.92)	5 (8.77)	0.577
Aspirin, *n* (%)	506 (99.80)	57 (100.00)	1.000
Statins, *n* (%)	488 (96.25)	56 (98.25)	0.694
Dapagliflozin, *n* (%)	117 (23.08)	18 (31.58)	0.154
Sacubitril Sodium Tablets/ACEI/ARB, *n* (%)	286 (56.41)	37 (64.91)	0.219
β-blockers, *n* (%)	427 (84.22)	52 (91.23)	0.161
Spirolactone, *n* (%)	17 (3.35)	2 (3.51)	1.000
Anticoagulant, *n* (%)	2 (0.39)	1 (1.75)	0.274
IRA-LCX, *n* (%)	63 (12.43)	9 (15.79)	
IRA-LAD, *n* (%)	244 (48.13)	38 (66.67)	0.008
IRA-RCA, *n* (%)	200 (39.45)	10 (17.54)	0.001
Pre-TIMI = 3, *n* (%)	99 (19.53)	7 (12.28)	0.184

BMI, body mass index; LAD, left atrium dimension; LVEF, left ventricular ejection fraction; TC, Serum total cholesterol; TG, Serum triglyceride; LDL-C, low-density lipoprotein cholesterol; HDL-C, high-density lipoprotein cholesterol; NT-proBNP, N-terminal pro B-type natriuretic peptide; hsTnT, high-sensitivity troponin T; eGFR, estimated glomerular filtration rate; MVO, microvascular obstruction; hs-CRP, highly sensitive C-reactive protein; LCR, lymphocytes to highly sensitive C-reactive protein; ESVI, left ventricular end-systolic volume to body surface area; EDVI, left ventricular end-diastolic volume to body surface area; ARB, angiotensin II receptor antagonist; ACEI, angiotensin-converting enzyme inhibitors; LCX, left circumflex branch; LAD, left anterior descending branch; RCA, right coronary artery; TIMI, thrombolysis in myocardial infarction. LGE, late gadolinium enhancement.

### Correlation between LCR and predictive indicators of LVT

As shown in Table 2, LCR was correlated with multiple indicators known to predict LVT, and was moderately correlated with peak hsTnT (*r* = −0.306, *p* < 0.001). It was weakly correlated with the peak value of NT-proBNP (*r* = −0.212, *p* < 0.001), LVEF (*r* = 0.19, *p* < 0.001), LGE% (*r* = −0.135, *p* = 0.001) and MVO% (*r* = −0.269, *p* < 0.001).

**Table 2:** Correlation between LCR and predictive indicators of LVT.

Parameter	Correlation Coefficient (r)	*p*-value
peak hsTnT, (ng/L)	−0.306	<0.001
Peak NT-proBNP, (pg/ml)	−0.212	<0.001
LVEF, (%)	0.19	<0.001
LGE%	−0.135	0.001
MVO%	−0.269	<0.001

LVEF, left ventricular ejection fraction; NT-proBNP, N-terminal pro B-type natriuretic peptide; hsTnT, high-sensitivity troponin T; MVO, microvascular obstruction; LGE, late gadolinium enhancement.

### Logistic regression analysis

As shown in Table 3, using univariate logistic regression analysis, we showed that LVT was correlated with Peak NTproBNP (*p* = 0.005), LVEF (*p* = 0.010), LGE% (*p* = 0.004), MVO% (*p* = 0.017), LCR (*p* = 0.001), IRA-LAD (*p* = 0.009). In multivariate logistic regression analysis, we found that LCR (*p* = 0.007), LGE% (*p* = 0.032), and IRA-LAD (*p* = 0.038) were significant predictors of LVT (Table 3).

**Table 3:** Logistic regression analysis.

Variables	Univariate logistic regression analysis		Multivariate logistic regression analysis	
	P	OR (95%CI)	P	OR (95%CI)
Male, *n* (%)	0.14	0.45 (0.16∼1.30)		
Age, (years)	0.936	1.00 (0.98∼1.02)		
BMI, (kg/m^2^)	0.607	1.02 (0.95∼1.10)		
Systolic blood pressure, (mm/Hg)	0.736	1.00 (0.99∼1.02)		
Diastolic blood pressure, (mm/Hg）	0.167	1.02 (0.99∼1.04)		
Heart rate, (Times/min）	0.559	1.01 (0.99∼1.03)		
PeakhsTnT, (ng/L)	0.410	1.12 (0.85∼1.48)		
PeakNTproBNP, (pg/ml)	0.025	1.43 (1.05∼1.96)		
TC, (mmol/L)	0.09	1.17 (0.98∼1.40)		
TG, (mmol/L)	0.739	0.96 (0.74∼1.24)		
LDL-C, (mmol/L)	0.495	0.88 (0.65∼1.19)		
HDL-C, (mmol/L)	0.191	1.66 (0.78∼3.53)		
Fasting blood glucose, (mmol/L)	0.141	1.07 (0.98∼1.18)		
eGFR, (ml/min/1.73 m^2^)	0.636	1.00 (0.99∼1.02)		
LGE%	0.004	1.02 (1.01∼1.03)	0.032	1.016 (1.001∼1.031)
MVO%	0.017	1.07 (1.01∼1.13)		
ESVI, (ml/m^2^)	0.458	0.99 (0.97∼1.01)		
EDVI, (ml/m^2^)	0.63	1.00 (0.98∼1.01)		
LCR	0.001	0.00 (0.00∼0.02)	0.007	0.001 (0.00∼0.123)
PLT	0.805	1.00 (1.00∼1.01)		
IRA-LAD, *n* (%)	0.009	2.16 (1.21∼3.84)	0.038	1.875 (1.035∼3.397)
Pre-TIMI, *n* (%)	0.189	0.58 (0.25∼1.31）		
Stroke, *n* (%)	0.224	0.48 (0.14∼1.58)		
Hypertension, *n* (%)	0.551	1.18 (0.68∼2.04)		
Smoking, *n* (%)	0.359	1.30 (0.74∼2.26)		
Diabetes, *n* (%)	0.077	1.69 (0.94∼3.02)		
Killip class ≥ 2, *n* (%)	0.4	1.53 (0.57∼4.11)		
LVEF, (%)	0.010	0.95 (0.91∼0.99)		

BMI, body mass index; LAD, left atrium dimension; LVEF, left ventricular ejection fraction; TC, Serum total cholesterol; TG, Serum triglyceride; LDL-C, low-density lipoprotein cholesterol; HDL-C, high-density lipoprotein cholesterol; NT-proBNP, terminal pro B-type natriuretic peptide, hsTnT, high-sensitivity troponin T, eGFR, estimated glomerular filtration rate; MVO, microvascular obstruction; hs-CRP, highly sensitive C-reactive protein; LCR, lymphocytes to highly sensitive C-reactive protein; ESVI, left ventricular end-systolic volume to body surface area; EDVI, left ventricular end-diastolic volume to body surface area; TIMI, thrombolysis in myocardial infarction; LGE, late gadolinium enhancement.

### Incremental value of LCR for LVT

**Figure 3:**
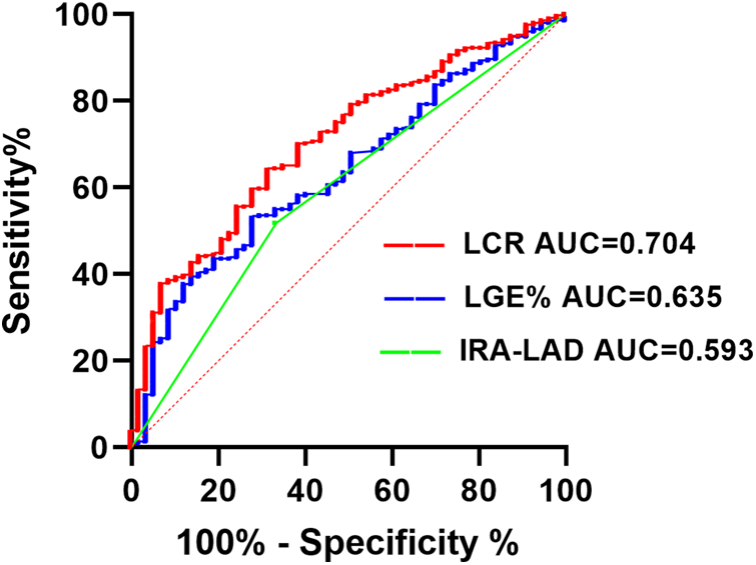
Receiver operating characteristic analysis (ROC) of LCR for identifying LVT. IRA, Infarct-related arteries; LAD, left anterior descending branch; LGE, late gadolinium enhancement; LCR, lymphocyte to C-reactive protein ratio; LVT, left ventricular thrombus.

ROC curve analysis was performed for factors associated with LVT identified in the multivariate logistic regression analysis. Figure 3 showed that LCR (AUC = 0.704, 95% CI: 0.638–0.769, *p* < 0.001) had a significant predictive value for LVT. The sensitivity and specificity for LCR predicting LVT in STEMI patients were 68.4% and 64.5%, respectively (Figure 3, Table 4).

**Table 4:** ROC curve analysis.

Variables	AUC	95% CI	P	Cut-off	Sensitivity	Specificity	Youden index
LCR	0.704	0.638–0.769	<0.001	0.0355	0.684	0.645	0.329
IRA-LAD	0.593	0.517–0.669	0.022	–	0.667	0.481	0.148
LGE%	0.635	0.565–0.765	<0.001	27.99	0.719	0.535	0.254

IRA, infarct-related arteries; LAD, left anterior descending branch; LGE, Late gadolinium enhancement; LCR, lymphocyte to C-reactive protein ratio; ROC, receiver operating characteristic; AUC, area under the curve; CI, confidence interval.

**Figure 4:**
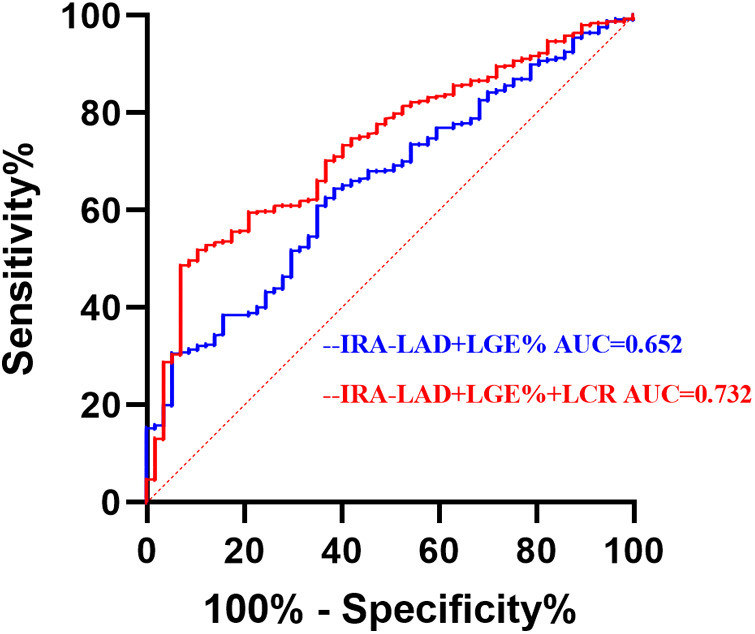
Receiver operating characteristic analysis (ROC) of combined parameters for identifying LVT. IRA, infarct-related arteries; LAD, left anterior descending branch; LGE, late gadolinium enhanced; LCR, lymphocyte to C-reactive protein ratio; LVT, left ventricular thrombus.

Based on multivariate logistic regression analysis, a traditional model including LGE% and IRA-LAD was constructed. Figure 4 shows that integrating the LCR into the traditional model improved the ability of LVT (AUC = 0.732, CI: 0.672–0.793, *p* < 0.001). Next, NRI and IDI were calculated.The results showed that the discrimination and reclassification accuracy of LVT was significantly improved by integrating the LCR into the conventional model (NRI 0.517, 95% CI 0.3087–0.7244, *p* < 0.001; IDI 0.030, 95% CI 0.0203–0.0397, *p* < 0.001), suggesting that integrating the integral LCR significantly improved the ability of the post-STEMI model to recognize LVT (Figure 4, Tables 5, 6).

**Table 5:** ROC curve analysis of combined parameters.

Variables	AUC	95% CI	P	Sensitivity	Specificity	Youden index
IRA-LAD + LGE%	0.652	0.585–0.720	<0.001	0.614	0.645	0.259
IRA-LAD + LGE%+LCR	0.732	0.672–0.793	<0.001	0.93	0.487	0.417

IRA, infarct-related arteries; LAD, left anterior descending branch; LGE, late gadolinium enhanced; LCR, lymphocyte to C-reactive protein ratio; ROC, receiver operating characteristic; AUC, area under the curve; CI, confidence interval.

**Table 6:** Discrimination accuracy and reclassification of LCR for LVT.

Models	NRI		IDI	
	Estimate (95% CI)	*P*-value	Estimate (95% CI)	*P*-value
Traditional model^a^	Reference	–	Reference	–
Traditional model + LCR	0.517 (0.3087∼0.7244)	<0.001	0.030 (0.0203∼0.0397)	<0.001

LCR, lymphocyte to C-reactive protein ratio; LVT, left ventricular thrombus.

^a^ Traditional model included LAD and LGE.

## Discussion

To the best of our knowledge, this is the first study to examine the relation between LCR and LVT in patients with STEMI. The main findings of this study are as follows: First, LCR was significantly associated with LVT in patients with STEMI. Second, lower ventricular volume was an independent risk factor for LVT in STEMI. Third, integrating LCR into risk models significantly improved LVT prediction.

STEMI is a leading cause of death globally (1, 2). Despite significant improvements in STEMI prognosis owing to the widespread use of early pPCI in recent decades, LVT remains a common complication. Previous studies have identified LVT as an independent risk factor for major adverse cardiovascular and cerebrovascular events (11). Although various clinical diagnostic tools exist for LVT, the risk of missed diagnoses remains. Therefore, identifying a reliable biomarker is crucial for early risk stratification of LVT.

Currently, LVT diagnosis primarily relies on imaging techniques, such as TTE, TTE with an ultrasound-enhancing agent, and CMR. TTE primarily detects LVT based on cardiac structure. However, small adherent LVTs, apically located LVTs, and cases with poor image quality may be missed. Although TTE has a high specificity (91%), its sensitivity is low (33%) (9, 11, 22, 23). TTTE with ultrasound-enhancing agent improves sensitivity to 60%, but over one-third of LVTs may still go undetected (24). RRecently, CMR, which detects thrombi based on histological characteristics, has become the gold standard for diagnosing LVT, with a sensitivity of 88% and specificity of 99% (10). In a study involving 265 patients with STEMI who underwent CMR within 3 weeks of onset, the incidence of LVT was 12.8% (25). Similarly, in this study, the incidence of LVT was 10.1% when CMR imaging was performed within a median of 5 d (4–6 d) of STEMI onset. However, studies have reported an overall LVT incidence rate of 2.7%–6.3% in patients with STEMI, likely due to the low sensitivity of TTE, which may lead to underdiagnosis (9, 22).

The correlation between inflammation and LVT has been confirmed by numerous studies (5, 6, 26). Recently, the LCR has been demonstrated to be a convenient and readily accessible inflammatory marker associated with the development and prognosis of various diseases (7, 13–17). IIt plays a crucial role in both the prognosis and diagnosis of cardiovascular diseases (19). In this study, we found that LCR was an independent risk marker for LVT after pPCI in patients with STEMI. Although the exact mechanism remains unclear, it may be related to several factors. Virchow's triad, which includes blood stasis, endothelial injury, and a hypercoagulable state, is the core mechanism underlying LVT after STEMI. Inflammatory factors are involved in Virchow's triad (27). Inflammatory responses play a crucial role in blood stasis during STEMI (28). During myocardial infarction-induced hypoxia, inflammatory cells are activated, producing large amounts of inflammatory factors that attach to endothelial cells, increasing blood flow resistance and contributing to blood stasis (5, 6, 22, 28). Simultaneously, the inflammatory cascade leads to endothelial injury after myocardial infarction, exposing the subendothelial tissue and collagen, which perpetuates inflammation and promotes a hypercoagulable state (22, 29). This may partially explain the results of this study. Furthermore, lymphocytes play a vital role in myocardial ischemia-reperfusion injury (29, 30). After reperfusion, lymphocytes rapidly accumulate at the site of injury, releasing cytokines and chemokines that attract other immune cells, such as neutrophils and monocytes, thereby exacerbating the inflammatory response (30). A previous study showed that a high neutrophil-to-lymphocyte ratio is associated with post-PCI hyperthrombosis in patients with STEMI; therefore, higher neutrophil counts or lower lymphocyte counts contribute to a higher incidence of high thrombotic load after PCI (31). CRP stimulates monocytes, endothelial cells, and smooth muscle cells to release tissue factors while inhibiting the release of tissue factor pathway inhibitors—a natural anticoagulant—by human endothelial cells, suggesting that CRP may have a prothrombotic effect. CRP may also increase LOX-1 expression, which plays a key role in the deleterious effects of oxidized LDL on endothelial function. Additionally, CRP enhances the proinflammatory effect induced by angiotensin II, further promoting thrombosis (32). Although this inflammatory response aids in clearing necrotic cells and tissues during the early stages, excessive inflammation can exacerbate myocardial damage and impair cardiac function recovery, thus significantly influencing the long-term prognosis of patients with STEMI (3, 29). CRP level is a powerful predictor of LVT after pPCI in acute anterior myocardial infarction (5, 6). ResultsIn a study of patients admitted to a cardiac intensive care unit, Litmanowicz et al. demonstrated that peak CRP levels predict ventricular thrombus formation (33). Additionally, Li et al. investigated infarct-related arterial thrombosis in patients with acute myocardial infarction and found that higher CRP levels were predictive of thrombus formation (34). As a combination of lymphocyte and CRP levels, LCR provides a more comprehensive reflection of the inflammatory state following STEMI. In this study, LCR correlated with peak high-sensitivity troponin T, peak N-terminal pro-B-type natriuretic peptide, late gadolinium enhancement percentage (LGE%), left ventricular ejection fraction, and microvascular obstruction percentage, all known risk markers for LVT (2, 35–37).This supports the findings of the present study from a different perspective. Although the correlation between LVT and other cardiovascular markers was not strong, a significant relation was observed.The relatively weak correlation could be because of the study's limited sample size or underlying mechanistic differences.

In previous studies, LGE% and infarct-related artery in the left anterior descending artery (IRA-LAD) were identified as LVT-related factors (36, 38). This study consistently found that LGE% and IRA-LAD were independent risk factors for LVT. Subsequently, a traditional risk model incorporating LGE% and IRA-LAD was established. Receiver operating characteristic analysis demonstrated that integrating LCR significantly improved the model's discriminatory ability for LVT (AUC = 0.732). When LCR was incorporated into the traditional model, discrimination and reclassification accuracy for LVT improved significantly (NRI = 0.517, IDI = 0.030, *p* < 0.001). This study demonstrates that LCR, as a simple and easily accessible clinical indicator, can predict LVT after myocardial infarction and enhance risk stratification. Therefore, LCR may serve as a valuable marker for identifying LVT in patients with STEMI undergoing pPCI.

### Limitations

First, this was a single-center retrospective study, which may have introduced unavoidable bias. Second, the sample size was small, as all included patients had STEMI and underwent CMR imaging during the acute phase. Nonetheless, this study highlights the additional diagnostic value of CMR for LVT. Third, the study population consisted solely of patients with STEMI; therefore, the findings may not be directly applicable to other patient groups. Fourth, in this study, the median time for CMR imaging was 5 (4–6) days after STEMI onset, which may have resulted in some LVT cases going undetected. The optimal time point for detecting the highest incidence of LVT remains unclear.

## Conclusion

We found that a lower LCR was independently associated with an increased risk of LVT in patients with STEMI after pPCI. Integrating LCR into the LVT risk model significantly improved its predictive accuracy.

## Data Availability

The raw data supporting the conclusions of this article will be made available by the authors, without undue reservation.
